# Effects of continuous positive airway pressure treatment on arterial stiffness and inflammatory factors in patients with coronary heart disease complicated with obstructive sleep apnea

**DOI:** 10.1186/s13019-024-03252-2

**Published:** 2025-01-11

**Authors:** Liang Wang, Yuanqi Wang, Tiantian Jiao, Linghao Xu, Endong Ji, Sakibur Rahman Tapu, Yehong Liu, Jiming Li

**Affiliations:** 1 Department of Cardiology, Shanghai East Hospital, School of Medicine, Tongji University, Shanghai, China; 2 School of Medicine, Tongji University, Shanghai, China; 3 Department of Emergency and Critical Care, Shanghai General Hospital, Shanghai Jiao Tong University School of Medicine, Shanghai, China; 4 Department of Cardiology, Shanghai East Hospital, School of Medicine, Tongji University, Shanghai, 200092 China

**Keywords:** Continuous positive airway pressure, Obstructive sleep apnea, Coronary heart disease, Arterial stiffness, Inflammatory factors

## Abstract

**Background:**

Continuous Positive Airway Pressure (CPAP) treatment brings more benefits than risks to most coronary heart disease (CHD) patients with obstructive sleep apnea (OSA). However, the pathophysiological mechanism by which CPAP treatment improves the prognosis of patients with CHD and OSA remains unclear. The purpose of this study was to clarify whether CPAP can improve arterial stiffness and inflammatory factor levels in CHD patients with OSA, and to further improve prognosis.

**Method:**

59 patients with coronary heart disease complicated by moderate to severe sleep apnea were divided into a CPAP treatment group (CPAP + coronary heart disease standard treatment) and a control group (only coronary heart disease standard treatment). Peripheral blood test reports were collected and pulse wave velocity (PWV) measurements were performed for each patient at the beginning, 3 months, and 6 months of treatment.

**Results:**

After 6 months of treatment, the CPAP group showed more significant improvement in the levels of inflammatory factors such as white blood cell (WBC), neutrophil (N), C-reactive protein (CRP), interleukin-6 (IL-6), procalcitonin (PCT), and PWV than the control group.

**Conclusion:**

After active treatment with CPAP, arterial stiffness and inflammatory cytokine levels in patients with coronary heart disease and OSA improved. This association should be given more attention in clinical practice, and sleep apnea should be actively treated.

## Introduction

Obstructive sleep apnea (OSA) is a common sleep respiratory disorder characterized by complete or partial obstruction of the upper airway during sleep, resulting in respiratory pauses or low ventilation, intermittent hypoxia, fluctuations in intrathoracic pressure, fragmented sleep, autonomic dysfunction, and inflammatory reactions [1, 2]. An increasing number of studies have shown that OSA is closely related to the occurrence and development of various cardiovascular diseases (CVD) and affects disease prognosis [3, 4].

Continuous Positive Airway Pressure (CPAP) therapy is an effective treatment for OSA [5, 6]. CPAP treatment brings more benefits than risks in most patients with CVD and OSA. Documents such as consensus or scientific statements at home and abroad recommend CPAP as the preferred treatment method.

Arterial stiffness is caused by dysregulation of elastin fibers and collagen, oxidative stress, mineral metabolism disorders, and low-grade inflammation [4], which can lead to increased myocardial preload and decreased coronary perfusion pressure. Arterial stiffness is a strong independent predictor of adverse cardiovascular events [5].

This study analyzed the clinical data of patients with coronary heart disease(CHD) complicated by OSA after CPAP treatment, observed the changes in inflammatory factors and arterial stiffness levels, and provided a clinical basis for the treatment of patients with CHD complicated by OSA.

## Materials and methods

### Clinical data

A total of 59 patients diagnosed with CHD were selected through coronary angiography at Shanghai Oriental Hospital from April 2022 to December 2023 and diagnosed with moderate to severe obstructive sleep apnea syndrome through a simple sleep monitor (ApneaLink™ Air. ResMed Inc). Among them, there were 45 males and 14 females, aged 63.3±10.1 years. The diagnostic criteria for obstructive sleep apnea syndrome are a sleep apnea hypopnea index (AHI) ≥ 5, and apnea is defined as a 90% decrease in airflow compared to baseline for at least 10 s; hypoventilation is defined as a 50% reduction in airflow for at least 10 s, accompanied by a 3% decrease in oxygen saturation or wakefulness [7].

The inclusion criteria were as follows: ① Age ≥ 18 years. ② Coronary angiography confirmed that one or more coronary arteries had ≥ 50% stenosis. ③ Moderate to severe OSA patients with AHI ≥ 15 were monitored using a simple sleep monitoring device (ApneaLink™ Air. ResMed Inc). ④ Informed consent was obtained from all the patients.

The exclusion criterion was: ① Clinical instability (hemodynamic or electrical instability). ② Severe renal insufficiency with EGFR < 30 ml/min. ③ Patients clinically diagnosed with acute myocardial infarction. ④ Treatment with systemic steroids or cyclosporine in the past 3 months. ⑤Active infection or major hematological, metabolic, or endocrine dysfunction. ⑥ Active malignant tumors that require treatment. ⑦ Pregnancy. ⑧ Persistent atrial fibrillation. ⑨ Severe aortic valve insufficiency or aortic valve stenosis. ⑩ Peripheral arterial disease (ankle brachial index) ≤ 0.9 or history of lower limb bypass surgery and/or endovascular treatment. ⑪ Severe pulmonary bullae or emphysema, severe chronic obstructive pulmonary disease, chronic respiratory failure. ⑫ Severe drowsiness with an ESS score greater than 15. ⑬ Previously received CPAP treatment. ⑭ Severe allergies.

All eligible patients who met the inclusion criteria signed informed consent forms. The Ethics Committee of Shanghai Oriental Hospital approved the study design and allowed the use of clinical data. All patients were divided into the CPAP treatment group (standardized treatment for coronary heart disease + CPAP treatment) and the control group (standardized treatment for coronary heart disease). All patients received the same standardized treatment for coronary heart disease (aspirin, clopidogrel, ACEI/ARB, beta-blockers, and statins), unless there were contraindications for these drugs.

### Data collection

General patient data were collected, including sex, age, hypertension history, diabetes history, smoking history, medication history, and family history. Fasting blood glucose, glycosylated hemoglobin, white blood cell count (WBC), neutrophil (N), N-terminal brain natriuretic peptide (NT-proBNP), fasting plasma glucose (FPG), glycosylated hemoglobin(HbA1c), total cholesterol (TC), triglyceride (TG), low-density lipoprotein (LDL), high-density lipoprotein (HDL), blood uric acid, blood creatinine, C-reactive protein (CRP), interleukin-6 (IL-6), procalcitonin (PCT), and other indicators. Each patient completed the Epworth Sleepiness Scale and underwent pulse wave velocity (PWV) testing at the initial treatment and at 3 and 6 months.

### Research methods

Patients who met the inclusion criteria were randomly divided into two groups: the treatment group, which received standardized treatment for coronary heart disease and used a CPAP ventilator (Philip Dorma 500 Auto. Philips Respironics.), and the control group, which only received standardized treatment for coronary heart disease. There were 40 patients in each group, and peripheral blood test reports were collected from each patient at the beginning, 3 months, and 6 months of treatment. The treatment group required each patient to be followed up at least once a month and encouraged the use of CPAP devices every night. During monthly follow-up, the usage rate of CPAP devices should not be less than 90%, and the average usage time should not be less than 4 h per night. At each follow-up, the software package including the CPAP device was used to calculate the average duration of CPAP use per night and AHI under CPAP treatment.

Among them, 18 patients in the treatment group withdrew from the study due to short CPAP usage time and low usage rate during the treatment period (requiring an average CPAP treatment time of more than 4 h per night and the usage rate should not be less than 90% every month ), and 3 patients withdrew from the study due to inability to follow up normally. There were a total of 19 effective cases in the treatment group. The average monthly CPAP usage rate (days of use/days of the month * 100%) is (93.1 ± 1.9)%, and the average CPAP usage time is 5.2 ± 0.8 h per night. Forty effective control cases were included. In total, 59 patients were included in both the groups.

**Fig. 1:**
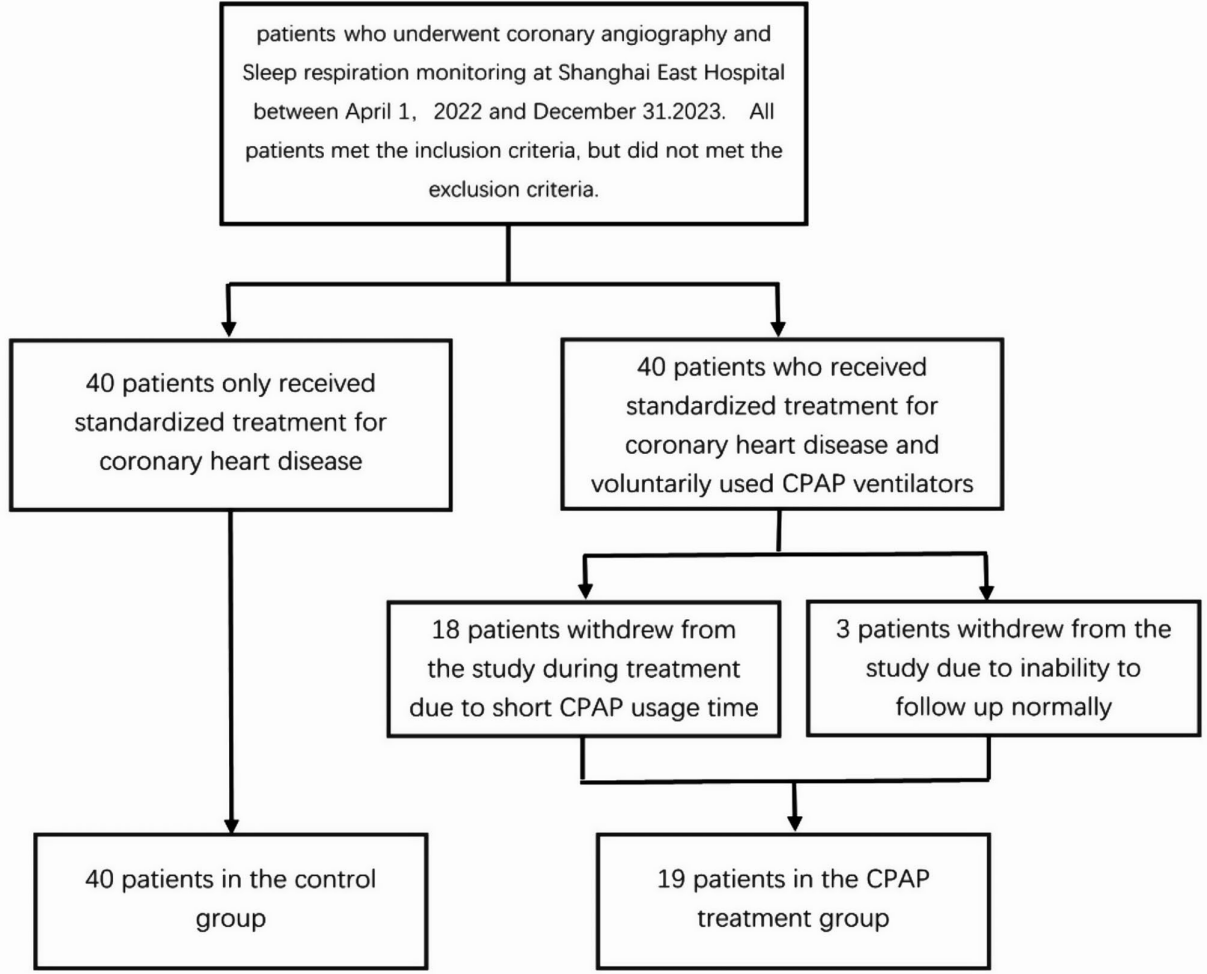
Study flow chart

Venous blood was drawn from all the enrolled patients on an empty stomach and sent to our laboratory for testing. Glucose oxidase technology was used to measure the FPG levels. Using existing enzyme technology, electrochemiluminescence technology has been applied to measure blood lipids and quantify IL-6 and PCT levels. All operations were strictly performed in accordance with the manufacturer’s instructions. PWV testing was performed using the Omron Atherosclerosis Tester (BP-203RPEIII. OMRON Inc) by the same physician in the resting state. The subjects and control group fasted overnight and quit smoking, drinking, and caffeine for at least 10 h before the vascular study (See Fig. 1).

### Statistical analysis

Statistical analyses were conducted using SPSS version 25.0. Count data are described as frequency (%), and continuous variable data are reported as median (IQR). The Shapiro–Wilk normal distribution test was used for data that conformed to a normal distribution, and an independent sample t-test was used for non-normal data. The independent sample Mann-Whitney U test was used for non-normal data. For all enrolled patients, the changes in inflammatory factors and PWV over time were represented by Δ. A high value between LPWV and RPWV was selected for PWV to evaluate the correlation between ΔPWV and changes in inflammatory factors. Pearson correlation analysis was used for correlation testing; *P* < 0.05 indicates a statistically significant difference.

## Results

Baseline data included baseline age, sex, hypertension history, diabetes history, smoking history, body mass index, glycosylated hemoglobin, fasting blood glucose WBC, N, CRP, IL-6, and PCT, and there were no significant statistical differences in blood lipids, renal function, PWV, and AHI (*P* > 0.05) (Table 1).

**Table 1:** Clinical feature of the study population at baseline

Clinical feature	the treatment group (*n* = 19)	the control group (*n* = 40)	*p*-value
Men, n (%)	16(84.2)	29(72.5)	0.327
Diabetes mellitus, n (%)	4(21.1)	13(32.5)	0.368
Smoking history, n (%)	13(68.4)	21(52.5)	0.065
Hypertension, n (%)	13(68.4)	21(52.5)	0.252
age	64(36–74)	66(39–78)	0.249
BMI(kg⋅m^− 2^)	24.2(19.3–30.4)	25(19-30.8)	0.326
MAP(mmHg)	102(82–115)	96.5(76–118)	0.197
HbA1c(%)	6(4.3–9.1)	6.1(5.2–9.8)	0.759
FBG(mmol/L)	5.68(4.31–12.65)	6.3(3.97–38.97)	0.215
TC(mmol/L)	4.64(3.13–5.93)	4.28(2.05–8.23)	0.074
TG(mmol/L)	1.55(0.67–3.82)	1.52(0.65–3.5)	0.565
HDL(mmol/L)	1.05(0.63-2)	0.99(0.67–1.74)	0.764
LDL(mmol/L)	3.07(1.56–3.95)	2.76(1.76–5.69)	0.303
WBC(*10^9/L)	9.14(4.53–11.8)	8.3(3.16–11.23)	0.127
N(*10^9/L)	6.26(3.11–9.95)	6.38(2.16–9.25)	0.236
CRP(mg/L)	3.34(1.6-13.57)	5.32(1.6-21.73)	0.221
IL-6(pg/ml)	12.58(3.67–91.94)	15.28(3.19–50.01)	0.44
PCT(ng/ml)	0.059(0.02–0.541)	0.165(0.013–0.99)	0.346
UA(µmol/L)	344.2(263.9-650.7)	358.4(231.6-519.4)	0.33
Cr(µmol/L)	77.5(50.7–206)	75(42.1-127.3)	0.833
LPWV(cm/s)	1428(1106–1719)	1406(1128–2013)	0.942
RPWV(cm/s)	1492(1133–1912)	1441(1157–1873)	0.501
AHI(t/h)	29(16–42)	27(16–51)	0.273

Compared with baseline data at three months, the CPAP treatment group showed significant statistical differences in the levels of other inflammatory factors, except for WBC, but no significant statistical differences were observed in PWV. At six months of treatment, except for WBC, all other inflammatory factors showed statistically significant differences, and PWV 、MAP decreased, which was statistically significant. (Table 2).

**Table 2:** Indicators of the treatment group after 3-mo and 6-mo

Indicators	baseline	3month	6month	*p*-value (3month vs. Baseline)	*p*-value (6month vs. Baseline)
LPWV(cm/s)	1428(1106–1719)	1458(1123–1723)	1279(1021–1543)	0.763	0.000**
RPWV(cm/s)	1492(1133–1912)	1485(1129–1882)	1286(1032–1679)	0.494	0.000**
WBC(*10^9/L)	9.14(4.53–11.8)	8.42(5.12–11.17)	7.88(5.83–9.67)	0.384	0.059
N(*10^9/L)	6.26(3.11–9.95)	5.33(2.91–8.23)	5.01(3.14–6.95)	0.015*	0.004*
CRP(mg/L)	3.34(1.6-13.57)	1.6(1.6–6.3)	1.6(1.21–5.1)	0.004*	0.003*
IL-6(pg/ml)	12.58(3.67–91.94)	5.29(2,28-16.7)	2.73(1.17–7.3)	0.000**	0.000**
PCT(ng/ml)	0.059(0.02–0.641)	0.04(0.01–0.29)	0.01(0.01–0.19)	0.000**	0.000**
MAP(mmHg)	102(82–115)	98(88–108)	94(84–102)	0.132	0.007*

*p < 0.05 ** *p* < 0.01

Compared with baseline data at three and six months, there were no significant changes in WBC, N, CRP, PCT, IL-6, or PWV in the control group, and the results were not statistically significant. (Table 3).

**Table 3:** Indicators of the control group after 3-mo and 6-mo

Indicators	baseline	3month	6month	*p*-value (3month vs. Baseline)	*p*-value (6month vs. Baseline)
LPWV(cm/s)	1406(1128–2013)	1405(1138–1987)	1398(1138–1980)	0.856	0.202*
RPWV(cm/s)	1441(1157–1873)	1458(1146–1882)	1436(1056–1890)	0.514	0.600
WBC(*10^9/L)	8.31(3.16–11.23)	8.02(4.27–9.82)	8.03(5.23–9.79)	0.173	0.793
N(*10^9/L)	6.38(2.16–9.25)	5.72(2.16–7.36)	5.78(3.34–7.55)	0.199	0.420
CRP(mg/L)	5.32(1.6-21.73)	4.72(1.6–68.8)	4.99(1.6–29.4)	0.191	0.935
IL-6(pg/ml)	15.28(3.19–50.01)	9.09(2,2-9.9)	5.75(1.53–20.1)	0.061	0.071
PCT(ng/ml)	0.165(0.013–0.99)	0.155(0.01–0.83)	0.145(0.01–1.35)	0.118	0.199
MAP(mmHg)	96.5(76–118)	96(84–112)	95.5(82–110)	0.317	0.123

Compared with the control group, the CPAP treatment group showed a significant decrease in WBC, PCT, and CRP levels after three months of treatment, with statistically significant differences. However, there was no statistically significant difference in IL-6 and PWV levels. After six months of treatment, the levels of CRP, IL-6, and PCT in the patient decreased significantly, and the results were statistically significant. The PWV of the treatment group patients showed a statistically significant decrease compared to the control group. (Table 4).

**Table 4:** Indicators of two groups after 3-mo and 6-mo

Indicators	the control group			the treatment group		*p*-value (3month)	*p*-value (6month)
	3 m	6 m		3 m	6 m		
LPWV(cm/s)	1405(1138–1987)	1398(1138–1980)		1458(1123–1723)	1279(1021–1543)	0.916	0.001*
RPWV(cm/s)	1458(1146–1882)	1436(1056–1890)		1485(1129–1882)	1286(1032–1679)	0.621	0.006*
WBC(*10^9/L)	8.02(4.27–9.82)	8.03(5.23–9.79)		8.42(5.12–11.17)	7.88(5.83–9.67)	0.042*	0.485
N(*10^9/L)	5.72(2.16–7.36)	5.78(3.34–7.55)		5.33(2.91–8.23)	5.01(3.14–6.95)	0.897	0.147
CRP(mg/L)	4.72(1.6–68.8)	4.99(1.6–29.4)		1.6(1.6–6.3)	1.6(1.21–5.1)	0.006*	0.000**
IL-6(pg/ml)	9.09(2,2-9.9)	5.75(1.53–20.1)		5.29(2,28-16.7)	2.73(1.17–7.3)	0.068	0.001*
PCT(ng/ml)	0.155(0.01–0.83)	0.145(0.01–1.35)		0.04(0.01–0.29)	0.01(0.01–0.19)	0.013*	0.000**

*p < 0.05 ** *p* < 0.01

**Fig. 2:**
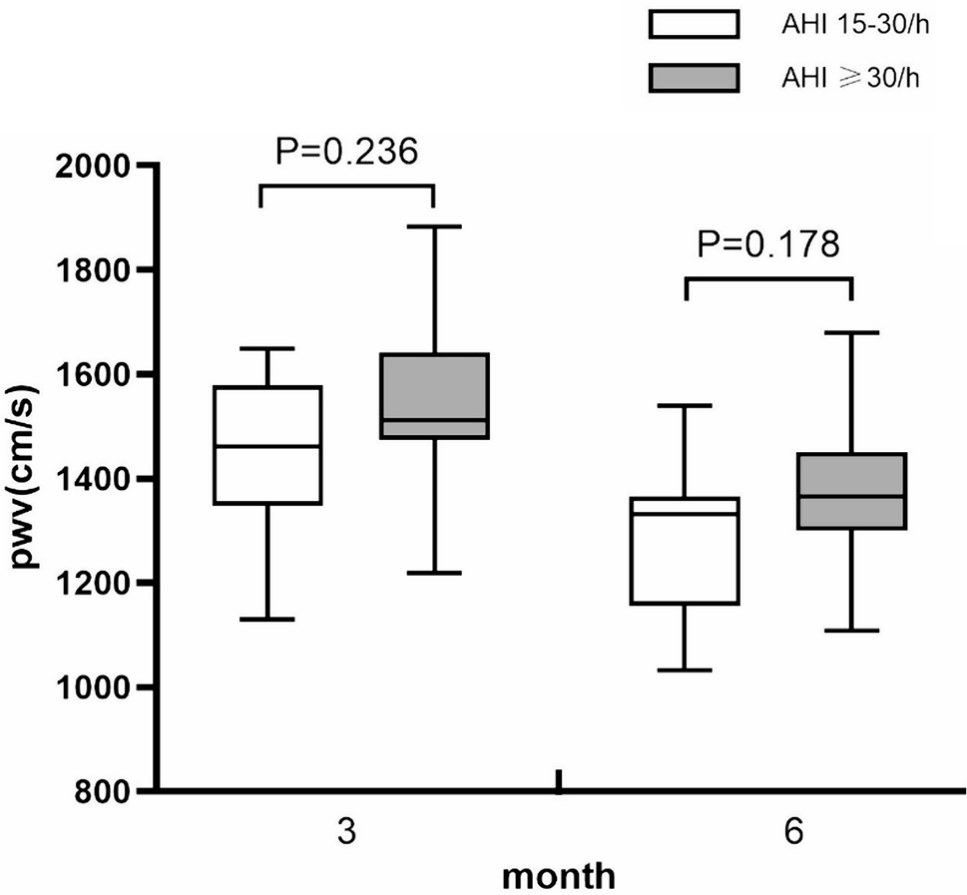
At 3 month and 6 mongth of treatment, there was no significant statistical difference in AHI 15–30/h group and AHI ≥30/h group

In the treatment group, there was no statistically significant difference in the improvement of PWV levels between patients with AHI ≥ 30 at 6 months of CPAP treatment and those with AHI levels of 15–30. (Fig. 2).

Correlation analysis was conducted between the levels of inflammatory factors and PWV in CPAP treatment group after six months. The results showed that there were no significant correlation between the decrease of inflammatory factors and the improvement of PWV, and the results were not statistically significant (Table 5).

**Table 5:** The relationship between ΔPWV and inflammatory factors

Indicators		*r* -Value	*p* -Value
ΔPWV(cm/s)	173(31–319)		
ΔWBC(*10^9/L)	0.86(0.06–4.53)	0.104	0.384
ΔN(*10^9/L)	1.56(0.03–5.5)	0.180	0.461
ΔCRP(mg/L)	0.4(0-11.97)	0.334	0.162
ΔIL-6(pg/ml)	8.27(0.94–88.84)	0.018	0.944
ΔPCT(pg/ml)	0.049(0.01–0.621)	0.173	0.507

## Discussion

OSA is characterized by frequent apnea and low ventilation during sleep, leading to blood oxygen saturation, sleep splitting, and daytime sleepiness. The common symptoms of sleep apnea syndrome are severe habitual snoring at night, accompanied by sleep apnea, frequent awakenings, anxiety and tension during awakenings, waking up early, feeling tired in the morning, experiencing significant drowsiness during the day, memory loss, personality changes, and abnormal exercise behavior. Sleep apnea syndrome can cause serious social problems, such as traffic accidents, and mental complications. Cardiovascular diseases, including coronary heart disease, arteriosclerosis, heart failure, arrhythmia, hypertension, stroke, and pulmonary arterial hypertension, are the most serious complications.

OSA is often combined with metabolic syndrome, and repeated nocturnal hypoxemia can exacerbate oxidative stress and inflammatory reactions, exponentially increase the risk of cardiovascular events and death [8–12]. In the Sleep Heart Health Cohort, after 8.7 years of follow-up, male patients aged 40–70 years with AHI ≥ 30 times/h had a 68% increased risk of developing coronary heart disease [13]. AHI is an independent risk indicator for predicting CVD mortality, and studies have shown that CVD patients with OSA have a 62% increased 5-year mortality rate compared with the control group [14].

The pathophysiological mechanism of CHD in OSA is currently unclear. Dursunoglu et al. [15] confirmed that sleep apnea promotes inflammation and thrombosis during atherosclerosis development. Respiratory obstruction occurs during sleep. Because of the role of breathing, the chest wall produces a large negative pressure, which increases the transmural pressure of the heart, leading to an increase in the afterload. At the same time, due to the increase in venous return, the increase in preload, and the congestion of the pulmonary circulation, hypoxia will lead to an imbalance of oxygen demand and supply to the myocardium, which will lead to angina pectoris and myocardial infarction.

Arterial stiffness is caused by dysregulation of elastin fibers and collagen, oxidative stress, mineral metabolism disorders, and low-grade inflammation [16], which can lead to increased myocardial preload and decreased coronary perfusion pressure. Arterial stiffness is a strong independent predictor of CVD and adverse cardiovascular events [17].

The main reason for increased arterial stiffness is adverse functional and structural changes in the vascular wall, including extracellular matrix degeneration; collagen deposition and cross-linking; elastin depletion and rupture; infiltration of vascular smooth muscle cells, macrophages, and monocytes; inflammation; and endothelial dysfunction [16]. An increase in arterial stiffness can reduce the compliance of the arterial system, leading to an increase in aortic systolic pressure and pulse pressure, thereby increasing the left ventricular afterload and myocardial load, resulting in left ventricular hypertrophy and increased myocardial oxygen demand [18]. In addition, an increase in forward pressure wave velocity caused by aortic sclerosis promotes the early arrival of reflected pressure waves during systole, resulting in a decrease in diastolic coronary artery perfusion pressure and myocardial oxygen delivery, leading to myocardial ischemia [19]. Therefore, an imbalance in the oxygen supply to the coronary arteries can lead to increased susceptibility to myocardial ischemia. In patients with CHD and OSA, this situation is particularly evident because of the multiple pathways involved in oxygen imbalance.

PWV is the most widely used measurement method for arterial stiffness (AS). It has strong prognostic value for predicting cardiovascular events and all-cause mortality. PWV is considered the “gold standard” for evaluating arterial stiffness owing to its simplicity, non-invasiveness, reproducibility, and proven predictive value in epidemiological and clinical studies.

Atherosclerosis in patients with CHD is not only a disorder of lipid accumulation and metabolism, but also that local or systemic inflammatory processes play a synergistic role in accelerating disease progression, ultimately leading to plaque rupture and clinical events [20]. Several studies have shown that inflammatory factors, such as high-sensitivity C-reactive protein and interleukin-6, are associated with cardiovascular risk [21, 22]. Therefore, based on previous studies, inflammation plays an important role in both OSA and arterial stiffness. This study also selected several commonly used inflammatory factors in clinical practice (WBC, CRP, N, IL-6, PCT) as the research subjects and observed the changes in inflammatory factors before and after different treatment regimens. Our study found that after six months of CPAP treatment, the levels of these common inflammatory factors were significantly reduced, indicating that CPAP may improve the prognosis of patients with CHD and OSA by reducing the inflammatory response.

CPAP is the first-line treatment for OSA, and its impact on arterial PWV has always been a concern. Vlachuantoni et al. [23] believed that CPAP treatment is an effective intervention to reduce arterial stiffness and has a positive impact on the survival rate of OSA patients with cardiovascular disease. However, the research results of Cardoso et al. [24] indicated that 6-month CPAP treatment can prevent further progression of the disease but cannot reduce aortic PWV. In addition, Galeneau et al. [25] conducted a long-term follow-up of OSA patients treated with CPAP for at least 4 years. The results showed that the increase in arterial stiffness after CPAP treatment was mainly related to primary cardiac metabolic disease. The study subjects were all patients with simple OSA, whereas our study subjects were patients with CHD complicated with OSA. The results showed that after 6 months of CPAP treatment for patients with coronary heart disease complicated by OSA, their PWV levels improved significantly. We further compared the PWV results of moderate OSA patients (AHI: 15–30) and severe OSA patients (AHI ≥ 30) after CPAP treatment and found no statistically significant difference between the two groups, indicating that CPAP is effective in improving arterial stiffness in patients with CHD complicated by moderate and severe OSA. This also suggests that CPAP treatment should be started in a timely manner for patients diagnosed with CHD complicated by moderate or severe OSA in clinical practice. However, the limitation of this study lies in the small number of patients in the CPAP treatment group, and further expansion of the sample size is needed for further research.

Shamsuzzaman et al. [26] found that plasma levels of high-sensitivity C-reactive protein were significantly elevated in patients with OSA and independently correlated with the severity of sleep apnea syndrome. Shinji [27] measured the plasma levels of inflammatory factors such as hypersensitive C-reactive protein(hs-CRP), IL-6, and TNF-α in 40 patients with sleep apnea syndrome and found that these inflammatory factor levels were correspondingly elevated in patients with sleep apnea syndrome. Our research found that both the experimental and control groups showed significant improvements in CRP, IL-6, and PCT levels after six months of treatment. However, compared with the control group, the decrease in CRP, IL-6, and PCT levels was more pronounced after CPAP treatment, indicating that CPAP can alleviate the levels of plasma inflammatory factors in patients with CHD complicated by OSA. Studies have shown that inflammation is involved in the occurrence of adverse events such as OSA or arterial stiffness. Therefore, we attempted to analyze whether there was a correlation between the improvement in PWV and the decrease in inflammatory factors in patients with CHD complicated by OSA after CPAP treatment. However, after correlation analysis, we found that there was no significant correlation between the decrease in CRP and the improvement in PWV, and more patients and longer follow-up are still needed to be included. This time point was further clarified.

### Study limitations

There are three main limitations in our study. First, The small sample size limited its power. especially in the CPAP treatment group. Second, The patient’s follow-up time is relatively short, only 6 months. Third, Inflammatory factors selected by the research institute may affect the experimental results if patients have potential bacterial infections and other microbial infections during sample collection.

## Conclusion

The relationship between CHD and OSA is clear, and arterial stiffness is an effective CHD predictor. Both OSA and arterial stiffness can lead to CHD through inflammatory pathways. Our research suggests that after active CPAP treatment, patients’ arterial stiffness and inflammatory factor levels improve, suggesting that more attention should be paid to this relationship in clinical practice, actively treating sleep apnea, and initiating CPAP treatment as early as possible. Eliminating adverse factors that affect the progression of coronary heart disease.

## Data Availability

No datasets were generated or analysed during the current study.

## Abbreviations

CPAP: Continuous Positive Airway Pressure
CVD: Cardiovascular diseases
CHD: Coronary heart disease
OSA: Obstructive sleep apnea
PWV: Pulse wave velocity
AHI: Apnea hypopnea index
EGFR: Estimated glomerular filtration rate
ACEI: Angiotensin converting enzyme inhibitor
ARB: Angiotensin receptor blocker
WBC: White blood cell count
N: Neutrophil
NT-proBNP: N-terminal brain natriuretic peptide
TC: Troponin, total cholesterol
TG: Triglyceride
LDL: Low-density lipoprotein
HDL: High-density lipoprotein
UA: Uric acid
Cr: Creatinine
CRP: C-reactive protein
IL-6: Interleukin-6
PCT: Procalcitonin
HbA1c: Glycosylated hemoglobin
MAP: Mean arterial pressure
